# Free latissimus dorsi musculocutaneous flap coverage of traumatic lower limb defect with transposition of the skin paddle: a flap within a flap

**DOI:** 10.1093/jscr/rjag652

**Published:** 2026-07-29

**Authors:** Marcus Jia-Ming Ng, Stephanie Li-Shan Chan, Ei Yadanar Khin, Vincent Khwee-Soon Tay, Jeremy Mingfa Sun, Christopher Wei Guang Ho

**Affiliations:** Department of Plastic, Reconstructive and Aesthetic Surgery, Changi General Hospital, 2 Simei St 3, Singapore 529889, Singapore; Department of Plastic, Reconstructive and Aesthetic Surgery, Changi General Hospital, 2 Simei St 3, Singapore 529889, Singapore; Department of Plastic, Reconstructive and Aesthetic Surgery, Changi General Hospital, 2 Simei St 3, Singapore 529889, Singapore; Department of Plastic, Reconstructive and Aesthetic Surgery, Changi General Hospital, 2 Simei St 3, Singapore 529889, Singapore; Department of Plastic, Reconstructive and Aesthetic Surgery, Changi General Hospital, 2 Simei St 3, Singapore 529889, Singapore; Department of Plastic, Reconstructive and Aesthetic Surgery, Changi General Hospital, 2 Simei St 3, Singapore 529889, Singapore

**Keywords:** latissimus dorsi, free flap, local flap, lower limb reconstruction, trauma

## Abstract

The free latissimus dorsi (LD) musculocutaneous flap is a reliable option in the reconstruction of traumatic lower limb defects. It is particularly useful in the coverage of extensive or circumferential wounds because of its large surface area and thinness. Our paper presents a modification of this free flap by performing perforator-guided transposition of the distal portion of the skin paddle and utilizing it as an additional local flap to cover exposed bone. We believe that this method is simple, reproducible, and useful in extensive wounds where the critical defect cannot be covered by the LD muscle alone.

## Introduction

Reconstruction of traumatic lower limb defects is a multidisciplinary team effort that aims to restore function and appearance of the affected limb [1]. Flap reconstruction is crucial when extensive tissue loss prevents direct closure or exposes vital structures such as bone, vessels, tendons, or implants [2]. Extensive lower-third leg defects often require free flap reconstruction because of inadequate locoregional soft tissue [3].

The advantages of using muscle flaps for lower limb reconstruction are well established. Firstly, they conform more easily to 3-dimensional defects, making them ideal for obliterating dead space and reconstructing irregular shaped wounds [4]. Secondly, muscle flaps possess rich vascular networks with lower vascular resistance compared to fasciocutaneous flaps [5]. Thirdly, the rich vascularity of muscle flaps also enables enhanced antibiotic delivery and bacterial clearance [5]. However, muscle flap harvest can result in functional impairment at the donor site [6]. Compared to fasciocutaneous flaps, muscle flaps are less able to tolerate weight bearing and require additional skin grafting, which is less durable and more prone to breakdown [7].

The free latissimus dorsi (LD) musculocutaneous flap is a well-published method for resurfacing extensive traumatic lower limb defects [3]. Its advantages include ease of harvest, large surface area, thinness, long and large-calibre vascular pedicle, versatility in flap inset and minimal donor site morbidity [8]. However, there are occasions where the large zone of the injury makes the muscle alone insufficient to cover all critical structures.

We present a modification of the free LD musculocutaneous flap for reconstruction of an extensive circumferential lower limb traumatic wound.

## Case report

**Figure 1:**
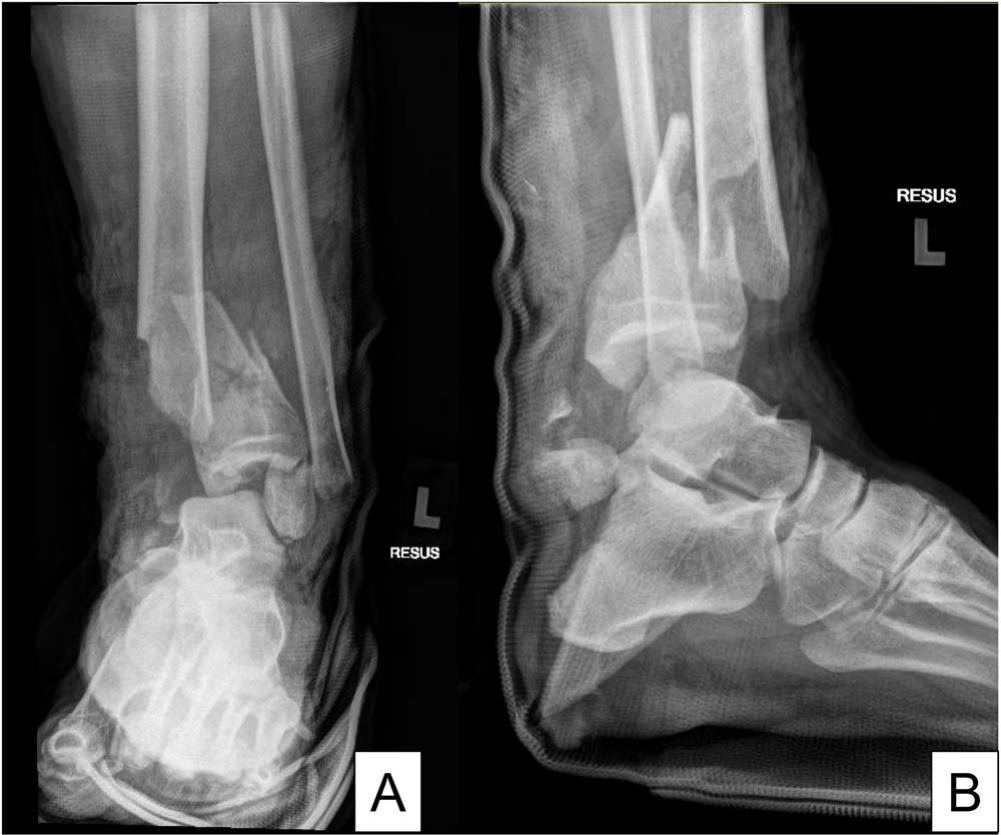
X-rays of the left ankle. (A) Anteroposterior and (B) lateral views.

A 46-year-old male motorcyclist sustained a Gustilo-Anderson type 3B fracture of the tibia and fibula, Weber type C ankle fracture (Fig. 1) and degloving injury of the left lower leg after a road traffic accident.

**Figure 2:**
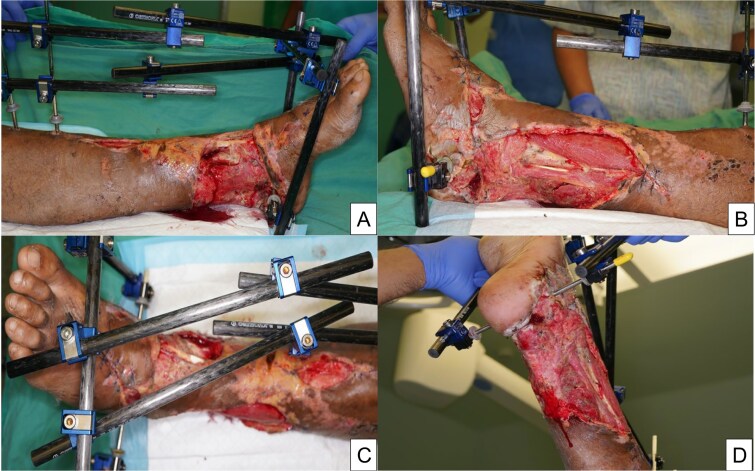
Left leg Gustilo-Anderson type 3B tibia-fibula fracture with near-circumferential skin degloving. (A) Medial, (B) lateral, (C) anterior, and (D) posterior views.

He underwent emergency external fixation of his fractures and serial debridement with negative tissue wound therapy. The resultant wound was extensive and near-circumferential (Fig. 2). The distal tibia (Fig. 2A) and fibula (Fig. 2B) was exposed at the medial and lateral ankle respectively, the proximal tibia was exposed at the upper third of the leg (Fig. 2C), and the tendon Achilles was exposed posteriorly (Fig. 2D). 17 days after presentation, he underwent fix-and-flap by the Orthopaedic and Plastic Surgery teams.

The free LD musculocutaneous flap was our first-choice for reconstruction due to the large surface area it provides. The contralateral side was used to facilitate an ergonomic two-team reconstruction approach, with concurrent flap harvest and preparation of the posterior tibial vessels with the patient in the left lateral position.

**Figure 3:**
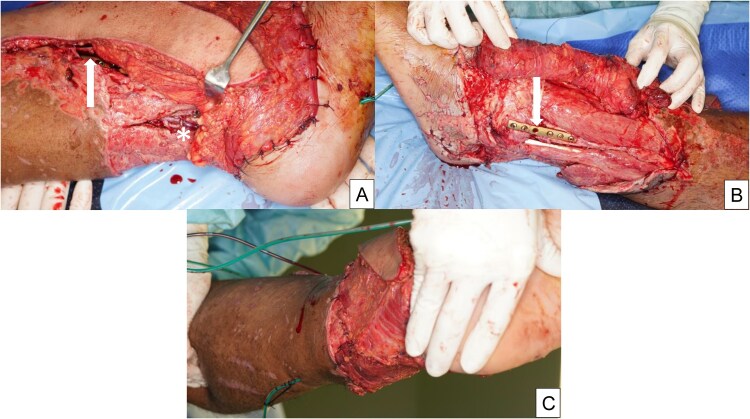
Internal fixation with titanium plate and locking screws and free latissimus dorsi musculocutaneous flap coverage. (A) Medial view showing the vascular anastomosis (*) and tibial implants (arrow), (B) lateral view showing the fibula implants (arrow), and (C) posterior views.

Open harvest of the right LD was performed with a 20 (length) ×7 (width) cm vertical skin paddle at the lateral edge of muscle, which permits a larger skin component to be harvested whilst still allowing for primary closure compared with a horizontal skin paddle. The LD tendon was transected to maximize thoracodorsal pedicle length. End-to-end micro-anastomosis to the posterior tibial vessels were accomplished with 9-0 Nylon (Fig. 3A). The flap was inset horizontally to achieve full wraparound coverage of the tibial (Fig. 3A) and fibular (Fig. 3B) implants as well as tendon-Achilles (Fig. 3C).

**Figure 4:**
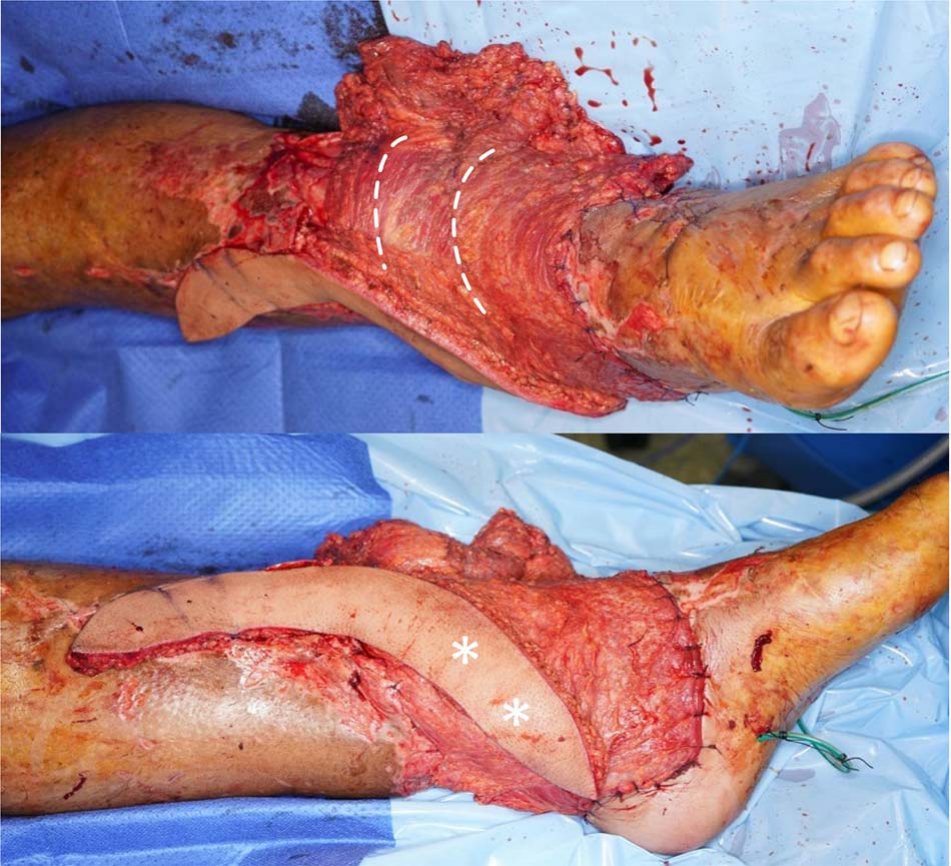
Transposition of the latissimus dorsi musculocutaneous flap skin paddle. (A) The original axis of the skin paddle is indicated by the pair of dotted white lines. (B) Dominant skin perforators (*) identified with Doppler ultrasound.

At this juncture, the proximal tibia at the upper leg remained uncovered. We decided to transpose the distal skin paddle (lying horizontally) perpendicularly to cover the exposed bone. A handheld doppler was used to locate the dominant musculocutaneous perforators. The distal portion of the skin paddle was elevated off the LD muscle, taking care to preserve the perforators and transposed 90° superiorly to cover the exposed proximal tibia (Fig. 4A), thus creating a flap (perforator-based transposition) within a flap (free). Intra-operative indocyanine green fluorescent angiography revealed satisfactory perfusion of the entire flap, including the distal skin paddle.

**Figure 5:**
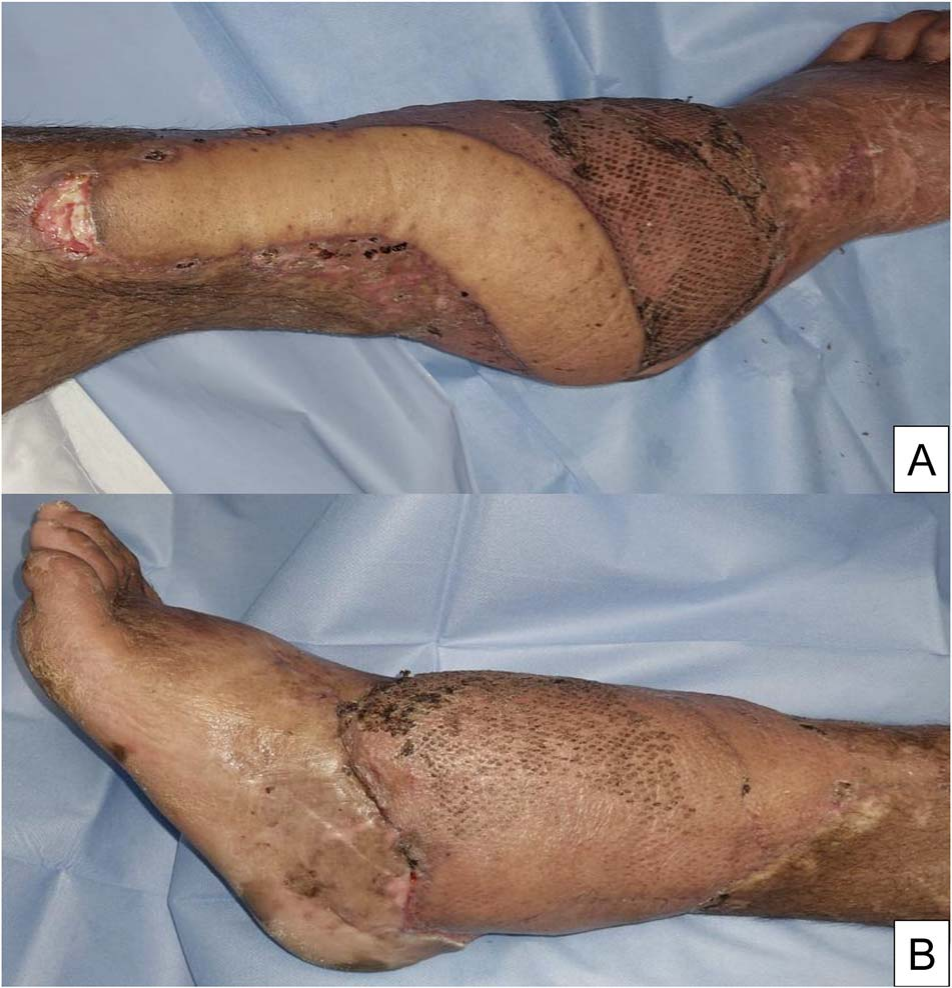
One month follow-up. (A) Medial view showing superficial dehiscence at the proximal skin paddle. (B) Lateral view showing satisfactory wound healing.

**Figure 6:**
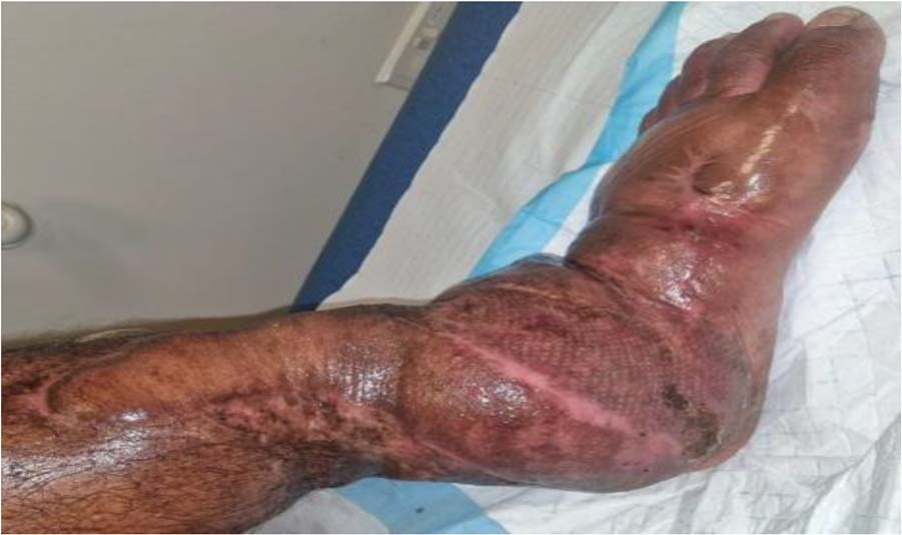
One year follow-up. Complete healing of wound.

A total of 2 weeks after free flap surgery, split thickness skin grafts were applied to the LD muscle. His recovery was unremarkable, with dressings applied to areas of superficial wound dehiscence (Fig. 5) until complete wound healing was attained (Fig. 6) at 3 months.

## Discussion

The LD free flap is a type 5 Mathes and Nahai flap with the dominant pedicle from the thoracodorsal system and secondary minor pedicles from the posterior intercostal and lumbar arteries [9]. There is a consistent perforator from the thoracodorsal artery 2 cm posterior to the lateral border of the LD and 8–10 cm inferior to the posterior axillary fold [10]. Our transposed skin flap was based on this perforator. The long vertical skin paddle sited at the lateral border of the muscle allows reach of transposed skin island beyond the muscle [11].

The large surface area of the LD muscle allows for an extensive coverage of wounds up to 25 × 35 cm and including circumferential defects. The skin paddle size can reliably reach 34 × 7 cm with primary donor site closure [12]. However, the LD muscle may still be insufficient in circumferential wounds with a long vertical defect as seen in this patient.

Traumatic wounds with exposed bone over the upper leg are challenging to reconstruct. One approach is to use local flaps but the thin skin with minimal laxity over the tibia limits the area for local flap harvest. Additional back grafting of the donor site may be required [13]. Local flaps may also lie within or adjacent to the zone of injury and have compromised perfusion [14]. Our technique obviates the need for additional local flaps and their associated disadvantages.

Another alternative involves serial procedures that may include bone burring, negative-pressure wound therapy, dermal matrix placement, and skin grafting [15]. This however increases costs, duration of hospitalization, and risks secondary osteomyelitis. In contrast, our method provides robust, single-stage coverage, and avoids skin grafting over a tenuous area.

Converting the distal skin paddle of a LD free musculocutaneous flap into a perforator-based transposition flap is a viable option when resurfacing extensive lower limb wounds that exceed the dimensions achievable with standard flap design. This technique can also be applied to other commonly used free musculocutaneous flaps (e.g. rectus abdominus and tensor fascia latae).
